# Living publications: How interactive artificial intelligence platforms are transforming research communication

**DOI:** 10.1002/ksa.70286

**Published:** 2026-01-28

**Authors:** Felix C. Oettl, Matthew Mudford, David Fendrich, Archontis Giannakidis, James A. Pruneski, Michael T. Hirschmann, Kristian Samuelsson, ESSKA Artificial Intelligence Working Group

**Affiliations:** ^1^ Department of Orthopedic Surgery, Balgrist University Hospital University of Zürich Zurich Switzerland; ^2^ Department of Orthopaedics, Institute of Clinical Sciences Sahlgrenska Academy, University of Gothenburg Gothenburg Sweden; ^3^ Sahlgrenska Sports Medicine Center Göteborg Sweden; ^4^ Tenfifty Gothenburg Sweden; ^5^ School of Science and Technology Nottingham Trent University Nottingham NG11 8NS UK; ^6^ Archimedes Unit in Artificial Intelligence, Data Science and Algorithms Athena Research Center Marousi Greece; ^7^ Department of Orthopaedic Surgery Tripler Army Medical Center Honolulu Hawaii USA; ^8^ University Department of Orthopedic Surgery and Traumatology Kantonsspital Baselland Bruderholz Switzerland; ^9^ Department of Clinical Research, Research Group Michael T. Hirschmann, Regenerative Medicine & Biomechanics University of Basel Basel Switzerland

**Keywords:** artificial intelligence, digital publishing, knowledge dissemination, natural language processing, scientific communication

## Abstract

Research communication is undergoing a paradigm shift. The traditional linear manuscript—foundational for centuries—increasingly reveals limitations in the digital era, struggling with information overload, delayed dissemination, and rigid formats. We propose a transition towards ‘living publications’: interactive, artificial intelligence (AI)‐enhanced platforms that evolve with new evidence. Unlike static papers, these systems utilise large language models (LLMs) and vector databases to interpret context, synthesise real‐time findings, and map interdisciplinary connections. This shift promises to democratise knowledge, accelerate validation, and enable dynamic evidence synthesis. However, it necessitates robust frameworks for verification, ‘version of record’ tracking, and peer review to maintain rigor. Successfully navigating this transition requires balancing technological innovation with preservation of academic values—ensuring that increased speed and accessibility enhance rather than diminish the quality of scientific discourse. As interactive platforms mature, they may potentially reshape how knowledge is shared, discovered, and applied, ideally accelerating scientific advancement through more dynamic, accessible, and collaborative research communication.

**Level of Evidence:**

NA.

**Abbreviations**
AI: artificial intelligence
IoT: internet of things
LLM: large language model
NLP: natural language processing

## 1 INTRODUCTION

Scientific communication may soon undergo a fundamental transformation, shifting from static manuscripts to dynamic, interactive platforms [12]. While open‐access models have improved the accessibility of research, they largely retain the limitations of the traditional PDF format: linear structure, fixed content, and a lack of interactivity [21]. In this commentary, we define ‘living publications’ not merely as digital documents, but as evolving ecosystems where artificial intelligence (AI) continuously integrates new data.

The current landscape relies on established structures that struggle to capture the complexity of modern research. Long review cycles and static formatting can render findings outdated by the time of publication [14]. In contrast, interactive platforms leverage Web 4.0 technologies—specifically AI and natural language processing (NLP)—to create spaces where researchers engage with content actively [21]. Rather than passively reading, users can query data repositories that update in real‐time [4]. This article outlines the conceptual architecture of these systems, the mechanisms of vector‐based retrieval, and the policy implications for peer review and citation standards in a post‐static era.

The concept of transforming scientific publishing for the digital era has a rich history predating current AI advancements. As early as 2012, Priem and Hemminger proposed ‘decoupling the scholarly journal’ [15], arguing that traditional publishing functions could be better served by specialised, interoperable platforms rather than bundled journal packages. Their vision presaged many current developments, including the separation of dissemination from evaluation and certification. Similarly, before the emergence of large language models (LLMs), Microsoft Academic Graph pioneered NLP‐driven approaches to scholarly information, noting that ‘the manners in which we conducted scholarly communications have changed dramatically’ [19]. They observed that while journal publications remained dominant, their traditional roles in dissemination and archiving were increasingly supplemented by ‘new breeds of online services’ including preprint repositories and collaboration websites offering faster, more accessible information.

This evolution has accelerated dramatically in recent years, with numerous platforms implementing aspects of this more interactive research paradigm. Tools like Elicit leverage AI to transform literature reviews from manual searches to question‐based explorations. Semantic Scholar enhances traditional search with semantic understanding of research content, while specialised platforms like Consensus and SciSpace focus on extracting and synthesising findings across papers. More recently, offerings like OpenAI′s Deep Research and Google′s AI co‐scientist demonstrate how LLMs can be applied to navigate and interpret scientific literature.

### 1.1 Current challenges with traditional research papers

The traditional research paper format, despite its historical significance, faces challenges in today′s digital landscape. These limitations are becoming increasingly apparent as research complexity grows and technology advances, creating a disconnect between how knowledge is generated and how it is communicated [12]. Information overload and accessibility barriers present known challenges. Researchers face an unprecedented volume of publications, making it difficult to thoroughly consume and synthesise relevant literature [2, 11]. The linear format requires readers to wade through extensive content to find specific information, already leading scholars to rely on abstracts or summaries rather than engaging with full manuscripts.

The static nature of traditional papers fundamentally constrains their utility in modern research. Once published, papers become fixed documents that cannot evolve with new findings or incorporate emerging perspectives. This immutability is particularly problematic in rapidly advancing fields where new discoveries might complement or challenge previous conclusions.

The current publication process itself presents additional obstacles. Long review cycles, often spanning months, can significantly delay the dissemination of important findings. This temporal gap between discovery and publication can render some research outdated before it reaches its audience, especially relevant in times like the COVID‐19 pandemic [12]. Moreover, the rigid structure of traditional papers often fails to capture the dynamic nature of modern research methods and results, particularly when dealing with complex datasets or interactive visualisations that could better illustrate findings.

### 1.2 The interactive platform paradigm

Fundamentally, interactive platforms will employ AI‐driven systems to understand and respond to user queries. Unlike traditional keyword searches, these systems utilise NLP to grasp the intent behind questions, enabling more precise and contextually relevant responses. This capability will allow researchers to navigate topics more intuitively, moving beyond the constraints of linear text to explore interconnected concepts and findings. LLMs can now interpret queries with unprecedented accuracy, considering context, user intent, previous searches, and domain‐specific terminology to deliver more meaningful results.

**Figure 1:**
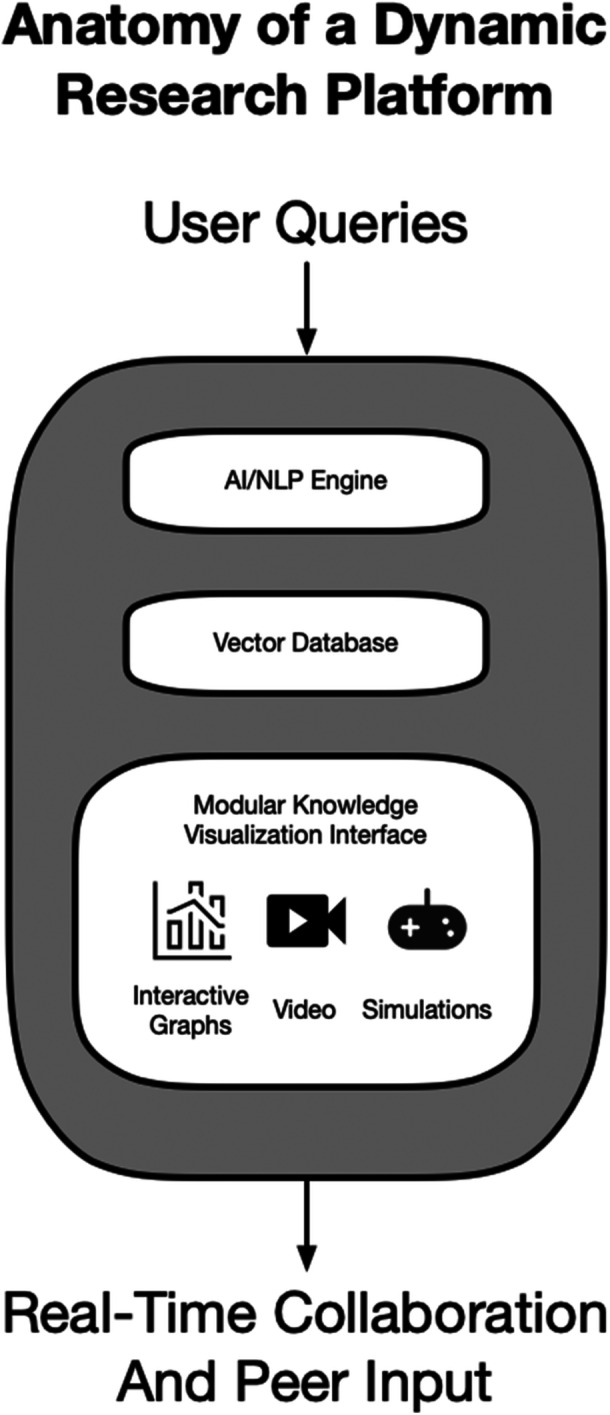
Anatomy of a dynamic research platform. AI, artificial intelligence; NLP, natural language processing.

**Figure 2:**
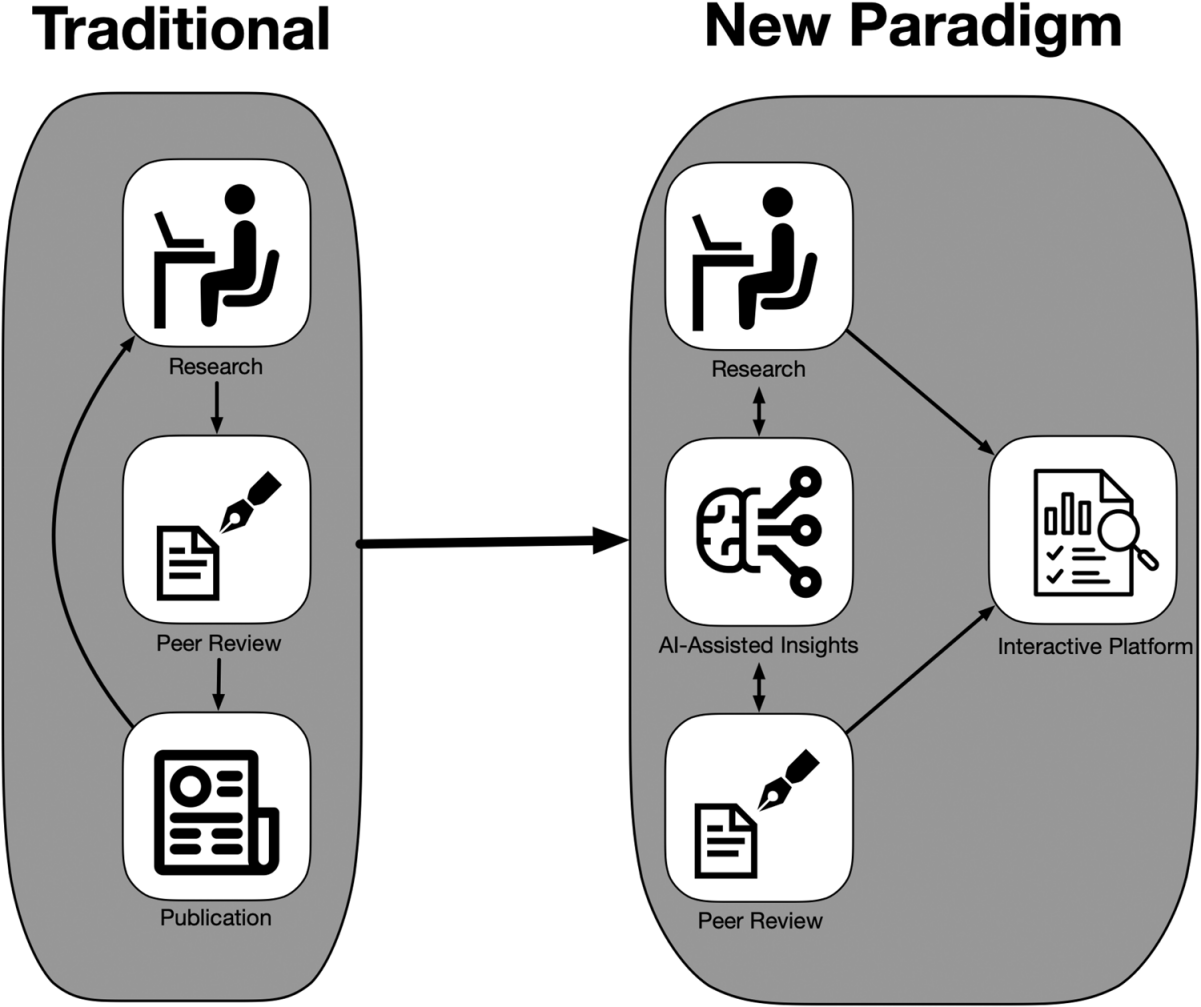
Schematic representation of the transition from traditional scientific manuscripts to dynamic, interactive platforms.

The dynamic nature of these platforms extends beyond search capabilities to content generation and presentation. By aggregating information from multiple sources, initially majorly peer‐reviewed literature, preprints, and verified datasets, these systems can synthesise comprehensive responses to complex queries. At the heart of current intelligent applications are vector databases, which store and retrieve data as high‐dimensional numerical representations rather than raw text (Figure 1). These specialised databases enable semantic similarity searches allowing systems to find conceptually related information regardless of keywords [18]. This integration enables researchers to quickly access relevant information across disciplines, fostering interdisciplinary connections that might have otherwise remained undiscovered. The platforms continuously update their knowledge base, incorporating new research findings and ensuring that users have access to the most current information in their field. Dynamic evidence synthesis may further enhance decision making in settings of limited or weak evidence. Through the automated aggregation of data and insights from individual studies published across multiple journals and databases, ‘living evidence synthesis’ may be attainable, which may eventually replace cross‐sectional systematic reviews and meta‐analyses [22]. Real‐time updates and cross‐referencing capabilities may further enhance the research experience. As new findings emerge, they are automatically integrated into the platform′s knowledge base, enabling immediate access to the most up‐to‐date research (Figure 2). This dynamic approach to content management ensures that researchers always work with the most current information available, while automated cross‐referencing helps identify relevant connections between different research areas and findings.

This paradigm is rapidly evolving beyond user‐initiated queries towards fully autonomous multiagent systems. Frameworks such as Microsoft′s AutoGen, Google′s AI co‐scientist, and open‐source initiatives like Robin are pioneering the creation of ‘virtual research teams’ [6, 7, 8]. In these systems, distinct AI agents, each with specialised roles—such as ‘Planner′, ‘Literature Reviewer′, ‘Data Analyst′, and ‘Writer′—collaborate to manage an entire research workflow, from hypothesis generation to final manuscript drafting. This represents a significant leap from interactive tools to semi‐autonomous systems capable of conducting complex research tasks with minimal human intervention, fundamentally changing the scale and speed at which scientific inquiry can be conducted.

Further pushing the boundaries of this paradigm is the move away from general‐purpose models towards dynamic and diverse ‘swarms’ of specialised AIs. Pioneering work demonstrates the power of ‘evolutionary model merging,’ a nature‐inspired approach where numerous smaller, open‐source models with specific capabilities are combined and ‘bred’ to create new, highly specialised models for niche tasks [1, 3, 9]. This method allows for the creation of a population of AI agents that can collectively improve and adapt over time, fostering a more sustainable and diverse AI ecosystem rather than the resource‐intensive process of training single, massive models from scratch [1, 10]. This approach could lead to research platforms that do not rely on a single intelligence, but on a constantly evolving collective of specialised intelligences.

### 1.3 Benefits and implications

The transition to interactive research platforms introduces expansive opportunities for knowledge democratisation and scientific advancement. Beyond simple information access, these platforms enable unprecedented levels of engagement from diverse audiences, including nonacademic practitioners, industry professionals, and policy makers [5]. This broadened participation has the potential to bridge the long‐standing gap between academic research and practical application, fostering a more dynamic ecosystem where theoretical insights can be rapidly tested and implemented in real‐world settings.

A particularly significant yet often overlooked benefit is the potential for real‐time research validation and replication. Interactive platforms can facilitate immediate peer verification of results, allowing researchers to quickly identify potential methodological issues or confirm findings across different contexts. This capability, combined with the platforms' ability to track how research is being used and interpreted across different fields, provides valuable meta‐analysis opportunities that can help identify emerging research trends and potential blind spots in current scientific understanding. These insights could fundamentally reshape how research priorities are determined and how funding is allocated across scientific disciplines.

The most immediate and transformative application of these agent‐based systems is the automation of systematic literature reviews. Research that has traditionally required months of manual, labour‐intensive effort can now be completed in a matter of hours [16]. Systems designed for this purpose can autonomously perform the entire review process, including literature identification, inclusion and exclusion filtering, data extraction, and the final compilation of the synthesised document [13, 16, 20]. This acceleration of evidence synthesis promises to be a watershed moment for evidence‐based practice in fields like medicine and policy, allowing researchers and practitioners to keep pace with the exponential growth of scientific literature and make decisions based on the most current findings.

### 1.4 Potential challenges and mitigation strategies

The transition to interactive platforms presents significant challenges that must be addressed to maintain the integrity of academic research. A fundamental concern is maintaining academic rigor in a dynamic environment—while the described platforms can facilitate faster information access and sharing, they cannot replace the deep analytical thinking and critical appraisal inherent in scientific discourse. Researchers must still engage in a thorough discussion of their findings, contextualising results within existing literature and identifying potential limitations. This critical thinking process, which forms the cornerstone of scientific advancement, cannot be automated or replaced by language models or AI systems in their current state.

Beyond the irreplaceable human element, key technical and institutional challenges must be addressed. These include ensuring data reliability through robust verification systems, developing sustainable funding models for platform maintenance, and creating effective archival systems. Additionally, clear guidelines for peer review in this new environment must be established by the academic community and frameworks for evaluating and crediting research contributions made through interactive platforms need to be developed. The successful implementation of these platforms will require a careful balance between innovation and preservation of academic values, ensuring that the speed and accessibility of new technology enhances rather than diminishes the quality of scientific discourse.

The advent of autonomous and multi‐agent systems introduces new challenges related to governance, ethics, and intellectual integrity. When AI agents can act, reason, and collaborate with limited human oversight, the risks move beyond simple misinformation to include autonomously generated yet entirely ‘hallucinated’ systematic reviews, the amplification of hidden biases at scale, and the potential for malicious use in generating fraudulent science. The ‘black box’ nature of complex agent‐to‐agent interactions makes auditing their scientific conclusions exceedingly difficult, raising critical questions of accountability and reproducibility. Furthermore, as these systems become capable of generating genuinely novel hypotheses, new frameworks will be required to address the attribution of discovery and intellectual property. Ensuring the responsible and ethical deployment of these powerful new tools is therefore a paramount challenge that requires immediate yet careful action from the scientific community.

The transformation from static papers to interactive platforms necessitates reconsidering not just how research is consumed, but also how it enters the scholarly ecosystem in the first place. Traditional publication processes centre around editorial offices and formal peer review systems that serve crucial gatekeeping, quality control, and credibility functions. An interactive platform paradigm must either replicate these functions or develop alternatives that maintain scientific integrity while leveraging new technological possibilities.

Emerging evidence suggests promising directions for such alternatives. Open peer review systems, where reviewer identities and reports are publicly accessible, are standard at various prominent AI conferences and have been implemented by several journals with encouraging results. Contrary to initial concerns, studies indicate that transparency does not compromise review quality, and may actually enhance accountability and constructive feedback [17]. Community‐based evaluation systems could further extend this approach, potentially allowing for continuous assessment rather than point‐in‐time reviews. Similarly, AI‐assisted tools could help address other editorial functions like plagiarism detection and methodological verification at scale, potentially with greater consistency than current approaches. The platform environment might also facilitate postpublication review and community annotations, creating living documents that reflect evolving scientific consensus rather than static snapshots of knowledge.

Another major benefit of interactive platforms is the transformation of publication incentives. By removing artificial space constraints, they could reduce the emphasis on novelty and positive results that contributes to publication bias. This might create more space for replication studies, null findings, and incremental work that does not gain visibility in prestigious journals but nonetheless contributes to scientific knowledge. However, developing credible alternatives to traditional peer review and editorial processes remains one of the most significant challenges for interactive platforms. Any sustainable solution will require careful collaboration between technologists, publishers, research institutions, and scientists themselves to ensure that new systems preserve the core values of scientific discourse while transcending current limitations.

## 2 CONCLUSION

The future of research communication may soon be transformed by interactive platforms that reshape how scientific knowledge is shared and consumed. These platforms will enable unprecedented levels of collaboration, while democratising access to research findings for both specialists and general audiences. The combination of natural language queries, real‐time updates, and AI‐driven personalisation may dramatically accelerate the pace of scientific discovery, allowing researchers to quickly find and build upon relevant work. This evolution towards more dynamic, accessible, and collaborative research communication will not only speed up the scientific process but also foster broader engagement with research findings, ultimately leading to more impactful and widely applied scientific discoveries.

## AUTHOR CONTRIBUTIONS

All listed authors have contributed substantially to this work: Felix C. Oettl performed literature review, and primary manuscript preparation. Editing and final manuscript preparation was performed by Matthew Mudford, David Fendrich, Archontis Giannakidis, James A. Pruneski, Michael T. Hirschmann and Kristian Samuelsson. All authors read and approved the final manuscript.

## CONFLICT OF INTEREST STATEMENT

Kristian Samuelsson is a member of the Board of Directors of Getinge AB (publ.) and medtech advisor to Carl Bennet AB. The remaining authors declare no conflicts of interest.

## ETHICS STATEMENT

The authors have nothing to report.

## Data Availability

Data sharing is not applicable to this article as no datasets were generated or analysed during the current study.
